# Cross-tissue omics-guided drug repurposing triangulates novel targetable mechanisms for Alzheimer’s disease and candidate genetic biomarkers for treatment stratification

**DOI:** 10.21203/rs.3.rs-7279662/v1

**Published:** 2025-08-05

**Authors:** Sathyaseelan Chakkarai, Michal Sadowski, Qiong Yang, Anushree Ray, Ruiqi Wang, Laurent F. Thomas, Raquel Puerta Fuentes, Mohsen Sharifi Tabar, Biqi Cui, Xueqiu Jian, Paul A. Nyquist, Dipender Gill, Allison E. Aiello, Thomas J. Montine, Syed Bukhari, Emily Rogalski, Steve E. Edland, Mary E. Haan, Claudia H. Kawas, Maria M. Corrada, Arash Salardini, Zsu-Zsu Chen, Jayandra Jung Himali, Claudia L. Satizabal, Robert E Gerszten, Bjørn Olav Åsvold, Myriam Fornage, Carlos Cruchaga, Mohamad Habes, Daniel I. Chasman, Lenore J. Launer, Margaret E. Flanagan, Ben Michael Brumpton, Marios K Georgakis, Noah Zaitlen, Agustin Ruiz, Stéphanie Debette, Sudha Seshadri, Muralidharan Sargurupremraj

**Keywords:** White matter hyperintensities, Dementia, Drug repurposing, pharmacogenomics

## Abstract

White matter hyperintensities (WMH) are covert magnetic resonance imaging (MRI) - markers of microvascular dysfunction and are primary vascular contributors to dementia, emphasizing its importance in prevention strategies. Here, we integrate gene expression and protein levels measured across plasma, cerebrospinal fluid (CSF), brain and multiple other tissues from population-based and biobank-scale data to triangulate druggable genes influencing WMH-burden and Alzheimer's disease (AD) and to map their spatial localization specifically in brain-cell types. Lowering the expression levels of *CALCRL, MAP3K11, PKN2* and *EPHB4* shows putative causal associations with reduced WMH-burden, and AD risk. These targets of clinically approved drugs, enriched in key cell types of the neurovascular unit and exhibiting cell-type specific effects in peripheral CD4 + T cell subsets, are implicated in regulating neuroimmune interactions and amyloid-β homeostasis. Gene-drug interaction analysis identifies higher levels of *GLTPD2* modifying the antidepressants associated increase in dementia risk contributing to a 73.3% risk reduction relative to the use of drugs. Furthermore, pharmagenic pathway studies identify the coagulation cascade as a targetable pathway associated with AD risk (HR: 2.23, 95% CI:1.85–2.69), providing orthogonal support to emerging therapies targeting coagulation components in treating neurodegenerative disorders.

## Introduction

As global life expectancy rises, dementia prevalence is projected to reach 75 million by 2030^1^, making prevention and delaying strategies a top public health priority. While therapies targeting aggregated forms of amyloid-beta (Aβ) mark a milestone in treating Alzheimer’s disease (AD) -the most common form of dementia - their benefit-to-risk ratio remains limited due to dose-dependent side effects, including amyloid-related imaging abnormalities and intracerebral hemorrhage^2^. Moreover, Aβ alone appears insufficient to explain the full spectrum of clinical symptoms. Most AD cases involve mixed pathologies that include cerebrovascular lesions^3^, particularly cerebral small vessel disease (cSVD), which often precedes neurodegeneration^4–7^. In recognition of this complexity, AD diagnostic criteria have expanded beyond a narrow focus on Aβ and tau pathology to incorporate vascular markers, including those of cSVD^8^.

WMH of presumed vascular origin - the most common magnetic resonance imaging (MRI) marker of cSVD - exhibit a dose-dependent relationship with both cognitive decline and dementia^6^. WMHs are also known to interact with neurodegenerative lesions^9^, potentiating the clinical expression of AD^10^. Notably, our recent works, using genetic instruments predictive of WMH burden and Mendelian randomization (MR) principles that leverages the natural randomization of genetic variation to effectively account for confounding and reverse causation; supports a putative causal link of larger WMH burden with increased AD risk^11,12^. Indeed, mechanisms closely related to WMH burden such as chronic cerebral hypoperfusion, have been shown to alter the equilibrium in Aβ clearance and exacerbate cognitive decline^13,14^, as demonstrated in the in vivo models^15^. Together, these findings position WMH as a promising therapeutic target for mitigating dementia risk, including AD.

Genomics-guided drug repurposing offers a powerful and pragmatic strategy for evaluating the effects of existing therapeutics on diseases beyond their original indications. Leveraging the MR framework, these studies assess the phenotypic consequences of genetic variants, specifically, single nucleotide polymorphisms (SNPs) located near protein-coding genes that encode known drug targets; thereby allowing to determine whether modulation of a given biological pathway is likely to reduce disease risk. Although genomics-guided drug repurposing strategies have begun to demonstrate their potential^16,17^, including our findings suggesting a protective effect of certain antihypertensive agents, such as β-blockers, on vascular pathways implicated in cerebrovascular disease;^18^ several challenges continue to limit their translational utility.

First, targeting established risk factors such as hypertension using antihypertensives presents inherent challenges; as its relationship with dementia varies with age and moreover genetic differences contribute to substantial inter-individual variability in treatment response^19,20^. Second, the success of genomics-guided drug repurposing depends critically on how accurately drug targets are mapped to biological pathways. However, many pathway knowledge bases incompletely capture drug-gene interactions, complicating the interpretation of genomic estimates and limiting their comparability to clinical trial outcomes^21^. Finally, the putative causal effect of the drug-targets on the disease risk may lack specificity due to “molecular-level (horizontal) pleiotropy”, where genetic instruments predictive of the molecular levels of a drug-target may also regulate multiple other targets, leading to off-target effects.

**Figure 1:**
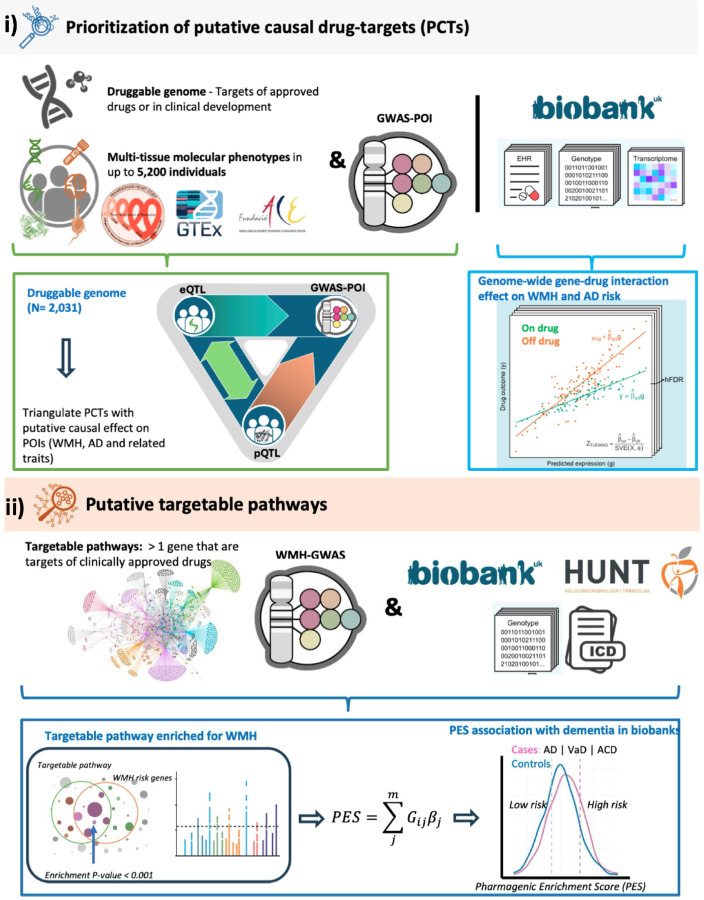
Study workflow. GWAS: Genome-wide association studies, POI: Phenotypes of interest, WMH: White matter hyperintensity burden, AD: Alzheimer’s disease, VaD: Vascular dementia, ACD: all-cause dementia, PES: pharmagenic enrichment score, QTL: quantitative trait loci, eQTL: expression QTL, pQTL: protein QTL.

To address these key challenges, we introduce a novel framework (Fig. 1) designed to systematically evaluate the repurposing potential of drug targets across the entire druggable genome. Unlike traditional approaches that rely on aggregated genetic effects across multiple targets, our framework captures individual drug-gene interactions, allowing for target-specific assessment for a given disease trait (e.g., WMH burden and AD risk), also mapping the biological pathways that are modifiable through pharmacological intervention. Importantly, it incorporates genetic heterogeneity to evaluate how individual differences influence drug response, advancing precision medicine strategies. A central feature of this framework is the integration of diverse molecular phenotypes (gene expression and protein levels) measured across multiple disease-relevant tissues, including brain and cerebrospinal fluid (CSF). This multi-layered analysis enables triangulation of therapeutic targets supported by molecular data that vary across time and tissue context. Furthermore, spatial mapping of prioritized drug-targets in post-mortem brain tissue provides insights into cellular localization, offering guidance for future drug delivery strategies.

## Results

### Prioritization of putatively causal drug-targets (PCTs) for WMH burden:

Of the 4,479 protein-coding genes from the druggable genome, we focused on 2,031 autosomal genes annotated as targets of approved drugs or drugs in clinical development (Supplementary Table 1). Using gene expression and genotype data from blood (Framingham Heart Study, FHS) and 17 additional disease-relevant tissues (Genotype-Tissue Expression project, GTEx), we fine-mapped SNPs with minor allele frequency (MAF > 5%) that function as proximal (cis) expression quantitative trait loci (eQTLs) and are putatively causal in regulating drug-target expression levels. By integrating these fine-mapped eQTLs with quantitative trait loci (QTL) association statistics for WMH burden from WMH-GWAS in a probabilistic framework, we identified an average of 1,634 drug targets across 18 tissue types that exhibited a non-zero gene-level colocalization probability (Supplementary Table 2), suggesting shared putatively causal variants regulating the drug-target expression and WMH burden. Given the possibility of QTL effects shared between the expression level of a given drug-target and WMH may be influenced by independent mechanisms unrelated to the drug-target of interest due to horizontal pleiotropy, that is not discerned through conventional colocalization approaches alone. Using Integration of TWAS and Colocalization Tool (INTACT) we integrated the colocalization evidence with the association strength (Z-score) of eQTLs with WMH burden (see Methods). By modeling gene expression as an intermediate variable between genetic variants and the complex trait and down-weighting gene-trait pairs with weak colocalization evidence, INTACT effectively identifies the genes causally mediating the WMH levels, distinguishing it from horizontal pleiotropy. Through this approach, INTACT prioritized 11 PCTs across 12 tissue types, each with a high posterior probability (> 0.90) of a causal relationship between their expression levels and WMH burden (Supplementary Table 3).

### Causal effect estimation and protein-level validation for prioritized PCTs across AD related traits:

**Figure 2:**
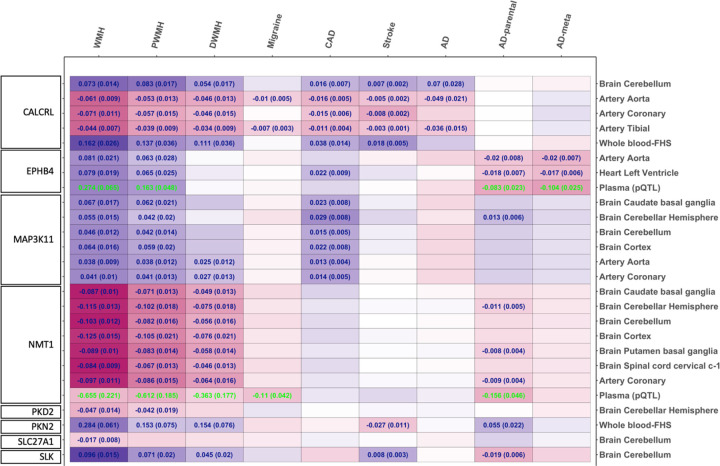
Causal effect estimation of prioritized PCTs with WMH and related traits in disease-related tissue types. Only showing PCT-trait pairs with significant MR association for WMH, corresponding causal effect estimates and (standard errors) with significant MR association for the other traits are annotated. Blue: eQTL-based estimates. Green: pQTL-based estimates. WMH: White matter hyperintensity burden; PWMH: Periventricular WMH; DWMH: deep WMH; CAD: Coronary artery disease; AD: Alzheimer's disease, AD-parental: GWAS based on parental history of AD, AD-meta: GWAS meta-analyzing AD-parental and clinically defined AD.

To quantify the causal effect sizes of the prioritized PCTs on WMH burden, AD risk, and related traits, we conducted MR analysis using carefully constructed genetic instruments. At the expression level, we used fine-mapped *cis*-eQTLs as instruments, further restricting to those that strongly predict PCT expression in relevant tissue types meeting standard instrumental variable (IV) assumptions. For protein-level analyses, we constructed the protein quantitative trait loci (pQTL) IVs by meta-analyzing three plasma-based pQTL datasets (see Methods). Each instrument predicted a specific PCT in a given tissue, and using the MR (Wald ratio) method, we found evidence of a putative causal relationship between 8 of the 11 PCTs and WMH burden (Fig. 2, Supplementary Table 4). Notably, expression changes in *CALCRL, NMT1, MAP3K11*, and *EPHB4* across multiple tissues showed putative causal effects on AD risk. While the direction of effects was largely consistent across tissue types, some targets (e.g., *CALCRL*) showed divergent associations, suggesting potential tissue-specific effects. Specifically, higher expression of *CALCRL* and *MAP3K11*, and lower expression of *NMT1*, in the brain tissues, were linked to larger WMH burden and AD risk. For *EPHB4* we observed discordance in the effect direction based on the outcome, as higher EPHB4 expression was associated with greater WMH burden but lower AD risk, a trend that is also reflected at the protein (pQTL) level. Colocalization analysis revealed that different and weakly linked (r^2^ = 0.09) pQTLs regulate EPHB4 protein levels for WMH (rs314337) and AD risk (rs909152) (Supplementary Table 5), explaining its differential relationship with larger WMH and reduced AD risk. Furthermore, of the 11 prioritized PCTs, two (*PKN2, SLC27A1*) were also found to have single-cell *cis*-eQTLs in peripheral blood mononuclear cell samples from the OneK1K cohort, which were used as instruments in MR analyses to determine cell-specific effects of the corresponding PCTs. Higher genetically proxied expression of *PKN2* in naive and central memory CD4 + T cells (CD4NC) was significantly associated with higher risk of AD (Supplementary Table 6). This association was directionally consistent with findings from bulk whole-blood data, further refining CD4NC as a relevant peripheral immune cell type linked to AD risk. Finally at the observed protein levels, higher *EPHB4* levels were associated with hippocampal atrophy - a key indicator of neuroinflammation and cognitive decline (Supplementary Table 7). No CSF-based pQTL association (*P* < 0.05) were observed for other available PCTs (*MAP3K11, NOS3, PKN2, EPHB4*) and no suitable pQTL instruments from brain proteomics were identified for the available PCTs.

### Phenome-wide association of the prioritized PCTs:

Next, we assessed the interaction for the prioritized 11 PCTs with drug compounds using Drug-Gene Interaction Database (DGIdb), which ranks drug-gene interactions based on the number and type of supporting sources - including regulatory databases (e.g., Food and Drug Administration [FDA] approved drugs), publications and expert-curated therapeutic knowledge bases (e.g., ChEMBL, Therapeutic Target Database [TTD]). Among the PCTs, *MAP3K11* received the highest interaction score (IS = 17.5), reflecting a specific interaction with the investigational compound CEP-1347 (Supplementary Table 8). For five PCTs (*EPHB4, GALK1, SLK, NOS3, PKN2*), the identified compounds were approved anti-neoplastic agents. For all PCTs except *CALCRL* and *ARNT*, the drug-gene interaction type was inhibitory in nature, where the binding of the compound decreased the expression levels of the PCTs. To further explore their functional impact, we investigated the loss-of-function (LoF) of these PCTs using phenome-wide association study (PheWAS) data from Genebass, a large-scale biobank resource of exome-wide association results. Specifically, focusing on the aggregate effect of LoF variants in a gene-based burden test scheme for the prioritized PCTs, revealed significant (*P* < 2.75E-06) associations with higher values for corneal integrity (targeting *MAP3K11*) and increased risk for kidney disease and related depression symptoms (targeting *PKD2*). We also observed sub-threshold associations (*P* < 1E-04) for increased white-matter integrity (targeting *ARNT*) and increased risk for digestive disorders (targeting *CALCRL*) (Supplementary Table 9).

### Pharmacogenomic determinants of treatment response to drugs interacting with PCTs:

Using genomic data linked to electronic health records (EHRs) from the UK Biobank (European-EUR), we studied for the interaction between the prioritized pharmacological compounds and genotype in identifying specific genes modifying the treatment response on WMH burden and dementia risk. More specifically, we performed TxEWAS (see Methods), testing for interactions between genetically predicted gene expression levels and medication use defined according to the ATC fourth level classification (see Methods). Of the 26 ATC classes with non-zero drug-PCT interaction scores (Supplementary Table 8), four classes (N06AX-other antidepressants, A07EA-corticosteroids, C01EB-cardiac preparations, and M04AA-uric acid production inhibitors) were sufficiently powered for the TxEWAS analysis (Supplementary Table 10).

**Figure 3:**
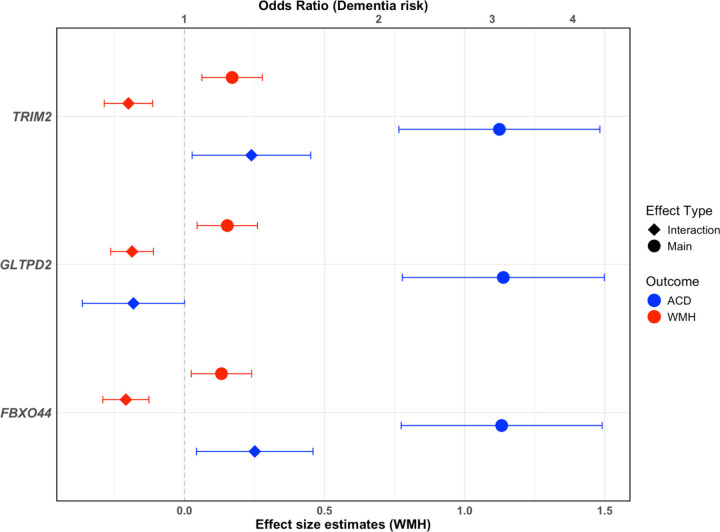
Gene-drug (Other antidepressants, N06AX) interaction effect and drug-main effect estimates for WMH burden and dementia risk. ACD: all-cause dementia, WMH: White matter hyperintensity burden

After hierarchical false discovery rate (hFDR) correction for the multiple tests across genes and tissue types; use of drugs from the N06AX-other antidepressants class was significantly associated with increased WMH burden for the main effects. However, gene-by-medication interaction analyses revealed that the higher genetically predicted expression of *TRIM2, FBXO44*, and *GLTPD2* in arterial and brain tissues significantly attenuated this medication-associated increase in WMH burden (Fig. 3, Supplementary Table 11a). Similarly, use of drugs from the N06AX-other antidepressants class was associated with a more than three-fold increased risk of dementia (Supplementary Table 11b). Notably, higher genetically predicted expression of *GLTPD2* modified this association (*P* = 0.05, hFDR corrected), reducing the disease risk by approximately 73.3% relative to the main effect of the medication use (Fig. 3, Supplementary Table 11b). Gene-by-medication interaction effects for the other ATC subgroups (A07EA, C01EB, M04AA), failed to survive the hFDR threshold.

Finally, recognizing that EHR-based analyses are susceptible to endogeneity bias - where individuals at higher baseline disease risk are more likely to receive treatment - we evaluated the main effects of genetically predicted gene expression levels on medication use, WMH burden, and dementia risk. No significant main effects were observed (Supplementary Table 11), suggesting that the identified gene-by-medication interactions are unlikely to be confounded by endogenous treatment effects.

### Regional and cell-specific expression patterns of PCTs in post-mortem brain tissues:

**Figure 4:**
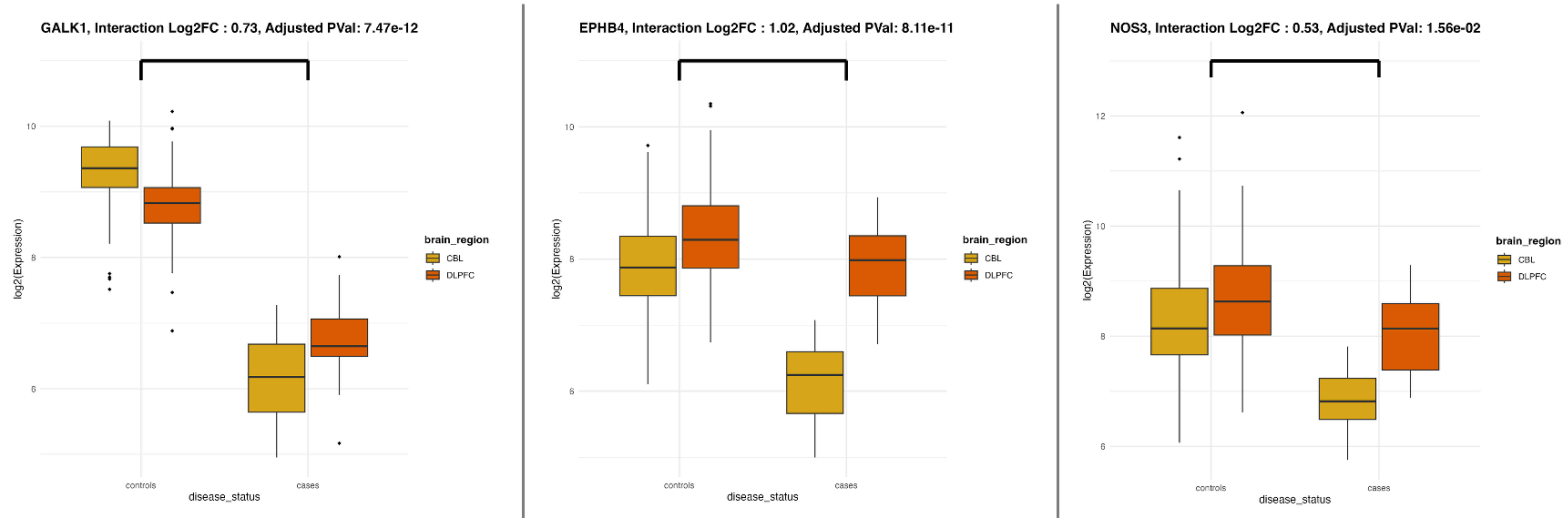
Region-specific (DLPFC vs cerebellum) differential expression of prioritized PCTs in post-mortem brain tissues of dementia cases and controls CBL: Cerebellum, DLPFC: Dorsolateral prefrontal cortex

Using bulk RNA sequencing data from post-mortem brain tissue samples (EUR) (dorsolateral prefrontal cortex [DLPFC] and cerebellum) of clinically and neuropathologically characterized dementia brain autopsy cases (N = 22) and matched brain autopsy controls (N = 148), we evaluated region-specific expression patterns of the 11 prioritized PCTs. Paired differential expression analysis was conducted using DESeq2, and log2 fold changes (Log2FC) were estimated for the main effect of disease status and its interaction with brain region. *GALK1, EPHB4*, and *NOS3* showed significant region-dependent differential expression, with higher expression in the DLPFC of dementia cases and more pronounced regional differences compared to controls (Fig. 4, Supplementary Table 12). We further assessed PCT expression at the cellular level using spatial transcriptomic data specifically from the DLPFC of SuperAging Study, the 90 + Study (EUR), focusing on fluorescently labeled cell types: endothelial cells (CD31), astrocytes (GFAP), and microglia (IBA1). Samples included cognitively normal individuals, and dementia cases with increasing cognitive impairment and chronic microinfarcts (mild cognitive impairment [MCI] with 2 microinfarcts, and dementia with 3 microinfarcts) (see Methods). Among the six PCTs with spatial transcriptomic data, pairwise Wilcoxon tests suggest elevated *EPHB4* expression in endothelial cells from MCI and dementia cases with vascular pathology compared to cognitively normal controls (Supplementary Fig. 1, Supplementary Table 13).

### Targetable pathways for WMH burden and their association with AD status:

**Figure 5:**
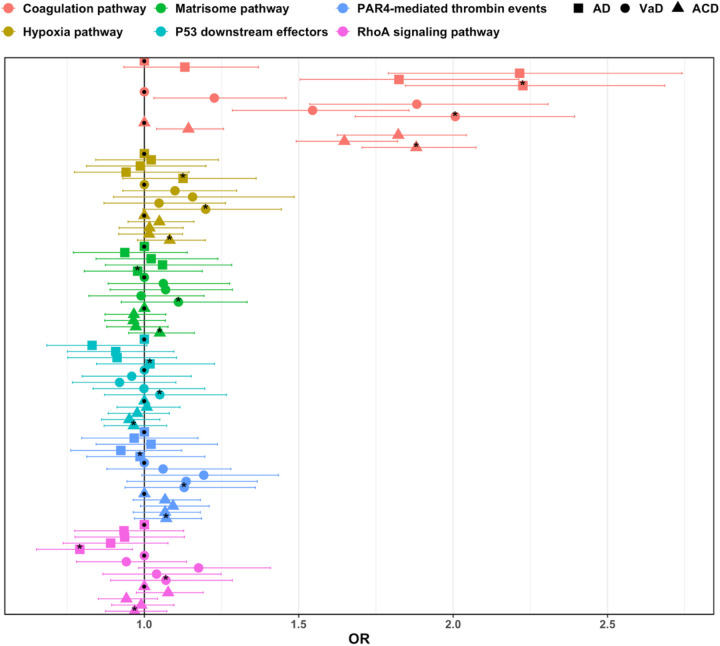
Targetable pathways enriched for WMH and their polygenic score (WMH-PES) association with dementia (AD, VaD, ACD) outcomes in UK Biobank. Color-coded denoting the representative pathway. Asterix (*) denote the association of the top PES bin vs the reference (•). AD: Alzheimer’s disease, VaD: Vascular dementia, ACD: all-cause dementia.

Using the pharmagenic enrichment score (PES) framework (see Methods), we investigated whether WMH-associated genetic variants cluster within biologically interpretable pathways that are potentially modifiable by existing pharmacological compounds. Gene-level association statistics for WMH were first computed by aggregating SNP-level effects across a range of GWAS p-value thresholds (P-value thresholding, *P*_T_), allowing us to capture varying levels of polygenicity. We then performed competitive gene-set enrichment analyses to test whether genes belonging to predefined, targetable pathways were more strongly associated with WMH than genes outside these pathways, while adjusting for gene-level confounders. This showed enrichment for seven targetable pathways for WMH (Table 1). The strongest association was observed for the hypoxia pathway (P value = 1.30×10^− 4^, β = 0.25, SE = 0.07, *P*_T_ = 0.005), followed by the thrombin signaling pathway mediated by the Protease Activated Receptor 4 (*P* value = 2.94×10^− 4^, β = 0.51, SE = 0.15, *P*_T_ = 0.50). Additional enrichment was seen in the coagulation cascade (*P* value = 4.80×10^− 4^, β = 0.15, SE = 0.04, *P*_T_ = 0.05). Pathways involved in cellular homeostasis (e.g., downstream effectors of p53 and the RhoA signaling pathway) and extracellular matrix maintenance also showed significant enrichment. Next, we sought to provide mechanistic insights of the enriched targetable pathways for WMH, by constructing PES at the individual level and studying their association with AD risk in independent, non-overlapping datasets of biobank scale and population-based data of EUR ancestry (UK Biobank-UKBB, Trøndelag Health Study-HUNT, Texas Alzheimer’s Research and Care Consortium-TARCC) and of non-European (Mexican-Americans) individuals (Sacramento Area Latino Study on Aging-SALSA). When PES values were stratified into quintiles, individuals in the top quintile for coagulation-related PES had a significantly increased risk of developing AD. In the UKBB, high PES for coagulation cascade was associated with more than a two-fold increased risk of AD (HR:2.23, 95% CI:1.85–2.69, *P* = 1.88×10^− 4^) (Fig. 5). This association was replicated in the HUNT dataset (HR: 1.99, 95% CI:1.74–2.26, *P* = 2.34×10^− 7^) and in TARCC with clinically ascertained AD cases (HR: 2.91, 95% CI:2.09–4.04, *P* = 0.03) (Supplementary Table 14). A similar trend was observed in the SALSA cohort of older Mexican-Americans (HR: 1.67, 95% CI: 0.86–3.23, *P* = 0.40), though it did not reach statistical significance, likely due to limited sample size.

## Discussion

In this study, capitalizing on genetic determinants reflective of changes in the molecular levels of drug-targets across diverse tissue types, we prioritize 11 drug-targets with putative causal effects on WMH, AD and related traits. Changes in the expression levels of *CALCRL, MAP3K11*, and *NMT1* display target-specific putative causal effects with a consistent effect direction with WMH, related traits and across the tissue types. We also observed a concordance in the effect direction at the protein level. The inhibitory effect was the most prominent interaction type between the prioritized drug-targets and pharmacological compounds. A phenome-wide scan assessing the loss-of-function of the genes encoding the drug-targets suggested better biomechanical properties of cornea and brain maturation markers while also identifying a potential increased risk of digestive complications (Crohn’s disease) and kidney disorders targeting certain candidates. Furthermore, studying the contribution of genetic variation to drug response across the genome, identified specific gene-drug interactions modifying the effects of the prioritized drug classes on WMH burden and dementia risk. Finally, pathways harboring targetable genes involved in cellular homeostasis and coagulation cascade were also enriched for larger WMH burden. Interestingly, higher WMH polygenic scores specific for the coagulation cascade pathway showed an association with higher AD risk, providing a pharmacological perspective from the genetic burden.

*CALCRL*, which encodes the calcitonin gene-related peptide type 1 receptor (CGRPr), is a key regulator of vasodilation and neuroinflammation within the cerebral vasculature. CGRPr is expressed in both glial and neuronal cells^22^, where their cell-type specific activation drives neurogenic inflammation, a mechanism central to migraine pathophysiology. Beyond migraine, CGRPr signaling has been implicated in pathological hallmarks of WMH such as cerebral hypoperfusion, blood-brain barrier dysfunction^23,24^, stroke^25^, and post-stroke functional outcomes^26^. In our study, we found a context-dependent or tissue-specific role of *CALCRL* expression, where its higher genetically predicted expression levels in brain tissues were causally associated with increased WMH burden, and to stroke and AD risk, highlighting a shared cerebrovascular mechanism. Notably, CGRPr antagonists that are approved for migraine treatment (e.g., erenumab, gepants) have demonstrated neuroprotective effects in AD mouse models, reducing amyloid-β deposition and tau pathology while enhancing cognitive performance^27,28^. Given their favorable safety profile and regulatory approval^29,30^, these agents represent promising candidates for testing repurposing potential in trials assessing brain structural changes. In particular, our findings provide a molecular rationale to investigate CGRPr blockade as a cerebrovascular-modulating therapy in populations with high WMH burden and cognitive impairment.

A common theme emerges from the other prioritized drug-targets (*MAP3K11, NMT1*), highlighting the role of post-translation modifications (PTMs) and cellular homeostasis in AD and its preclinical processes. *MAP3K11*, primarily expressed in neurons, encodes mixed-lineage kinase-3 (MLK3) that modulates the MAPK signaling cascade through phosphorylation, promoting apoptosis in response to metabolic stress. MLK3 inhibitors have shown neuroprotective effects in vivo and are currently being evaluated in clinical trials for multiple neurodegenerative disorders^31^. Our observation of lowered *MAP3K11* expression levels with reduced genetic liability to AD risk, in conjunction with in vitro studies suggesting MLK3 modulation in reducing amyloid-β induced toxicity^32^, place MLK3 inhibitors in a pertinent context with AD. This association also extended to a lower WMH burden, suggesting that targeting MLK3 may be particularly beneficial in preclinical stages of AD. Additionally, the development of highly selective, brain-penetrant MLK3 inhibitors capable of crossing the blood-brain barrier is particularly promising^33^, as application of such compounds in in vivo studies have been shown to reduce inflammatory cytokine levels and decrease cerebral infarct volume in a dose-dependent manner^34,35^. Given that several MAPK pathway inhibitors are already in development for oncologic indications^36^, and that anti-cancer drug exposure has been linked to lower AD risk^37^, repositioning MLK3 inhibitors could represent a novel therapeutic strategy. Our findings also highlight *NMT1* (N-myristoyl transferase-1) as a novel candidate, where reduced expression was associated with both lower WMH burden and decreased AD risk. NMT1 catalyzes N-myristoylation, a lipid-based PTM that is relatively underexplored in AD^38^. Its relevance to endocytosis pathways and regulation of amyloid-β peptide processing^39,40^ makes it an intriguing target for further study. Finally, spatial gene expression studies support a context-dependent role particularly for *EPHB4* - encoding an ephrin receptor and key regulator of vascular remodelling and angiogenesis. *EPHB4* is disproportionately elevated in the DLPFC of AD cases and in the endothelial cells from the brain sections of individuals with MCI and dementia, highlighting additional key molecular markers orchestrating the shared mechanisms between vascular dysfunction and neurodegeneration.

Mounting evidence suggests that antidepressant use may contribute to adverse neurocognitive outcomes, yet the underlying mechanisms and moderating factors remain incompletely understood. Our findings add a genomics perspective to this discourse by demonstrating that individuals prescribed N06AX class other antidepressants - which encompasses drugs prescribed for a range of affective disorders, including major depressive disorder, post-traumatic stress disorder, postpartum depression, and insomnia - exhibit an elevated WMH burden and heightened dementia risk. These observations align with prior epidemiological studies linking antidepressant exposure to cognitive decline, cSVD, and dementia risk^41–45^. Since depression is a prodromal feature of dementia that prompts antidepressant use, associations between antidepressants and dementia risk are attributable to confounding-by-indication or reverse causation due to shared mechanisms^46^. However, the consistent association between antidepressant exposure and accelerated cognitive decline and dementia risk^42,43^, across diverse antidepressant classes (SSRIs, tricyclics, and other antidepressants) suggests a shared intrinsic property that may be causally linked to neurodegenerative processes. Moreover, the cognitive and affective consequences observed upon treatment discontinuation, such as worsening cognitive performance in dementia-free individuals^43,47^ and exacerbation of depressive symptoms in dementia patients^48^, underscore how balancing the risk of accelerated cognitive decline with the therapeutic benefits of these medications remains a clinical conundrum that requires personalized approaches. Our pharmacogenomic analysis identifies *TRIM2, FBXO44*, and *GLTPD2* as potential genetic modifiers, whose higher expression levels attenuate the deleterious effects of N06AX antidepressants on WMH burden, with *GLTPD2* also reducing dementia risk. *GLTPD2* encodes a glycolipid transfer protein involved in sphingolipid trafficking, essential for maintaining endothelial cell function and vascular integrity^49,50^. Perturbations in sphingolipid metabolism have been linked to cardiovascular and cerebrovascular injury, larger infarct volumes, and post-stroke outcomes^51–53^. Additionally, a recent multi-omics study, identifies key members of the sphingolipid pathway (sphingomyelins) whose dysregulation mediates AD pathogenesis^54^ and that administration of a sphingolipid receptor modulator alleviated synaptic plasticity and cognitive impairment in amyloidogenic mice models^55^. Together, these findings advance a model in which *GLTPD2*-mediated regulation of sphingolipid homeostasis mitigates the neurovascular and cognitive consequences of antidepressant exposure and highlight the potential utility of pharmacogenomic stratification to inform safer, more effective antidepressant prescribing.

Shared genetic susceptibility studies suggests that nearly one-third of the genetic risk for WMH burden is not mediated by conventional risk factors, pointing toward additional and potentially targetable pathophysiological mechanisms^11^. By focusing on pathways composed of genes encoding targets of known pharmacological compounds, our study identified significant enrichment for WMH burden in biological processes related to hemostasis, vascular integrity, endothelial function, and angiogenesis. Although disruptions in these processes are known to play a central role in the pathophysiology of cSVD^56,57^, our findings extend this understanding by providing a genetic burden perspective linking vascular dysfunction with neurodegenerative outcomes. Indeed, abnormalities in cerebral blood flow and BBB integrity that are central to WMH pathophysiology, are among the earliest detectable brain changes in the progression from MCI to late-onset AD^58,59^. Specifically, we observed individuals with a higher polygenic burden in coagulation-related pathways showing more than a two-fold increased risk for AD. This provides additional support to the observations from experimental AD models and population-based studies implicating activation of the coagulation cascade and vascular injury in promoting neuroinflammation and cognitive decline^60–62^. This process also appears to be further complicated by maladaptive vascular regeneration, such as non-productive angiogenesis, which destabilizes the microvasculature at sites of injury^63^. Notably, fibrinogen deposition in the brain parenchyma has been shown to trigger pathogenic microglial activation, induce pro-inflammatory pathways, and lead to dendritic spine loss and cognitive deficits^60^. In this context, our observation that the association between coagulation pathways and AD risk is evident only in the presence of elevated WMH burden re-emphasis that fibrinogen-mediated pathology may be region-specific and dependent on the degree of BBB dysfunction^60^. Moreover, the pathway-specific genetic susceptibility linking vascular dysfunction and neurodegeneration suggests that these individuals are most likely to benefit from targeted interventions and their identification would be of clinical importance. Notably, therapeutic strategies aimed at neutralizing the inflammatory form of fibrin without disrupting systemic hemostasis have shown promise in suppressing innate immune-driven neurodegeneration in AD mouse models^64,65^, supporting our findings.

Our study offers a systematic framework and tractable roadmap for drug-target prioritization within the druggable genome by integrating multi-layered evidence from population genomics, transcriptomics, and proteomics. By combining causal inference models, pathway-specific polygenic enrichment, and spatially resolved gene expression analyses, we identify shared molecular drivers that potentially mediate the earlier observed causal association between cerebrovascular dysfunction and dementia risk^12^. Importantly, our findings extend beyond traditional biomarker discovery by highlighting actionable targets, several of which are already modulated by pharmacological compounds with established safety profiles, thus offering candidates for designing clinical trials and mechanistic validation in established AD models. Moreover, the diversity of implicated biological processes (e.g., neuroinflammation, sphingolipid metabolism, and cellular hemostasis) underscores the heterogeneous pathophysiology, which likely manifests in region-specific ways across the brain. These observations argue against one-size-fits-all approaches and reinforce the need for precision medicine strategies in tackling the multifactorial nature of AD. Nonetheless, several limitations warrant attention. First, the estimated causal effects reflect lifelong exposure, necessitating consideration of age-specific dynamics^66^. Second, although our analyses integrate multi-tissue and spatial molecular data, including brain tissue, as well as single-cell molecular data that resolve cell-type specific effects primarily in blood, the limited availability of brain single-cell data (N < 500)^67^ restricts our ability to thoroughly investigate brain cell-specific effects. This limitation highlights the importance of future collaborative efforts aimed at increasing sample sizes^68^, to enable more robust QTL-based drug-target prioritization frameworks. Third, the relative scarcity of proteomic data, compared to transcriptomic resources, poses a significant challenge for cross-modal validation. Although WMH served as the primary imaging phenotype, our prioritization strategy can be extended to other imaging features of vascular brain aging, such as perivascular spaces, which are relevant for amyloid-beta clearance^69^, and other established markers of neurodegeneration. Finally, expanding these analyses to include non-European populations will be essential for generalizability, as diverse genetic architectures become better represented in global datasets.

## Online methods

Figure 1 provides an overview for i) prioritizing PCTs and ii) identifying targetable pathways.

### Exposures:

Genetically predicted gene expression levels and protein levels of the druggable genes were considered as the exposure measure. The druggable genes include autosomal and protein-coding genes (N = 2,031) that are annotated as targets of approved drugs or drugs in clinical development^70^ (Supplementary Table 1). We leveraged gene expression and eQTL data from the FHS (N = 5,257, plasma) and multiple-other tissues from the GTEx project (N = 838)^71^. Cell-specific cis-eQTL data from peripheral blood mononuclear cells were obtained from the OneK1K cohort (N = 982)^72^. For the PCTs prioritized at the expression level, we performed additional validation at the plasma protein level from FHS plasma proteomics data profiled using 7K SomaScan assay (N = 956, see Supplementary Methods) and using pQTL information from both publicly available plasma-based pQTL datasets^73–75^, and through collaboration for brain and CSF pQTL datasets (Spanish Fundacio-ACE cohort [N = 1,186]; Knight-ADRC [N = 1,537]).

### Outcomes:

For the outcomes in our PCT prioritization scheme, we used European-only association statistics from the largest GWAS of WMH, related traits, and clinically diagnosed AD. We also included AD GWASs based on parental history of dementia from the UK Biobank, and that comprised both clinically diagnosed cases and broader definitions of AD using self-reported parental history as a proxy for dementia diagnosis (Supplementary Table 15). To identify targetable pathways, we assessed the association of pathway-specific polygenic scores with dementia outcomes (AD, Vascular dementia, VaD; all-cause dementia, ACD) in large-scale biobank data from European ancestry populations. Dementia status in the UK Biobank (cases = 7,203; controls = 15,247) and the HUNT (cases = 3,385; controls = 20,147) was determined using ICD-9 and ICD-10 codes, as described in the Supplementary Methods and elsewhere^12^. Additional replication was pursued in a population-based cohort of European (TARCC, cases = 754; controls = 602) and Hispanic ancestry (SALSA, cases = 79; controls = 658), where dementia (AD) diagnosis was based on the NINCDS-ADRDA criteria and Modified Mini-Mental State Examination (3MS), respectively.

### Statistical Analysis:

#### PCT prioritization

*Data preparation* - Using genotype and gene expression counts from the FHS, we conducted a probabilistic fine-mapping (DAP-G)^76^ of eQTLs that are putatively causal in regulating the expression level of a specific drug-target. DAP-G computes, i) a gene-level posterior inclusion probability (PIP) for harboring at least one causal variant and ii) a variant-level PIP that is causal for the gene expression. We considered only common variants within 500Kb of the gene boundary, adjusting for age, sex, and cohort-specific variables. Additionally, we considered pre-computed fine-mapping results from the GTEx multi-tissue data (v8). Second, the fine-mapping results from DAP-G along with the SNP-WMH effect estimates from the largest WMH GWAS^11^ were used to estimate i) the association statistic (Z-score) of the fine-mapped eQTLs with WMH in a transcriptome-wide association study (TWAS) framework^77^, and ii) the gene-level colocalization probability for shared causal variants between gene expression and WMH levels^78^. *PCT prioritization* - To address confounding from eQTLs regulating multiple drug-targets due to linkage disequilibrium (LD), we used INTACT - a probabilistic integration method^79^. INTACT combines the TWAS association statistic and gene-level colocalization probability using a shrinkage parameter, excluding eQTL-gene pairs lacking colocalization evidence due to weak LD. This approach is known to better meet the IV assumption of no pleiotropic effect between the gene expression and trait levels (exclusion restriction)^79^. An INTACT-posterior probability greater than 0.9 was considered indicative of the presence of a putative causal effect between drug-target and WMH. *PCT and pharmacological compound interaction* - For the prioritized PCTs, first, we tested their interaction with known pharmacological compounds using the DGIdb (www.dgidb.org)^80^. DGIdb aggregates drug-gene interactions from several curated sources, including regulatory databases (e.g., FDA) and expert-curated knowledgebases (e.g., Pharmacogenomics Knowledge Base (PharmGKB), Drug Target Commons). It harmonizes drug and gene records linking them to supporting evidence from publications records to create comprehensive interaction entries, including the interaction types (inhibitors, antagonist, etc.). Interactions are scored based on the number of unique sources and references. Second, we used Genebass^81^ to perform a PheWAS-based lookup assessing the effects of loss-of-function (LoF) variants in the prioritized PCTs. Genebass provides gene-based association statistics for rare coding variants across multiple annotation categories - including LoF - spanning 4,529 phenotypes in 394,841 UK Biobank participants. We applied a significance threshold of *P* < 2.75E-06, correcting for the number of PCTs tested and the median gene-based significance level (P = 5E-06) reported in Genebass.

#### Causal effect estimation for the prioritized PCTs and validation

*Mendelian randomization analyses* - We estimated the putative causal effect of the prioritized PCTs on WMH and other related traits (Supplementary Table 15). Only fine-mapped eQTLs from the DAP-G analysis and satisfying the instrumental variable definition (LD independent - r^2^ < 0.1)^82,83^ were considered as instruments (Supplementary Table 16). For cell-specific analyses, as described earlier^84^, we used single-cell cis-eQTLs reaching a P-value threshold of < 1×10^− 5^. A Wald-ratio MR method was used for PCTs, with only one eQTL instrument predicting their expression levels^85^. Effect alleles of the eQTL instruments were defined as the allele associated with an increase in the corresponding gene expression values. *pQTL level validation* - We validated the concordance in the causal effect direction of prioritized PCTs using pQTLs predicting protein abundance. pQTL instruments were constructed from a fixed-effect meta-analysis of multiple publicly available blood-based pQTL datasets (N = 49,568) of European ancestry with the protein levels quantified using the SomaScan platform^73–75^, using METAL. Additional brain and CSF-derived pQTLs from the Knight-ADRC (N = 1,537) and Spanish Fundacio-ACE cohort (N = 1,186) were also considered. To ensure adequate instrument strength while maintaining the causal estimation accuracy, and as evidenced in several studies^86,87^, a larger p-value threshold (*P* < 0.001) was used in defining the pQTL instruments. Only LD-independent SNPs (r^2^ < 0.1) within 1 Mb of the PCT transcription start site were considered. Causal effect estimates were obtained using the inverse-variance weighted (IVW) method and pleiotropy-robust MR methods (MR-RAPS, MR-Egger, weighted median). Consistent estimates in the IVW and at least two pleiotropy-robust methods indicated a causal association between protein-abundance of the PCTs and WMH and other related traits^88^. Colocalization analysis ruled out additional pleiotropic effects due to LD^89^, with a posterior probability (PP4) ≥ 75% indicating evidence for a shared causal variant. Causal estimates in both eQTL and pQTL based analyses were scaled to represent 1 standard deviation (SD) change for the gene expression and protein levels. Analyses were performed using R version 3.3.2 and the TwoSampleMR package. *Protein level validation* - Using the FHS participants from the Offspring cohort (N = 956) with plasma protein levels (7,596 aptamers, 7k SomaScan assay v4.1) and MRI data, we tested the association of the protein levels of the prioritized PCTs with WMH and hippocampal volume (HV) - a key indicator of neuroinflammation and cognitive decline, using linear mixed effects models, adjusting for age at MRI, sex, total intracranial volume, time difference between blood draw and completion of MRI, blood sample batch, and plate. Familial correlation was accounted for using random effects correlated according to degree of relatedness. Protein levels of each aptamer were log-standardized to reduce skewness. Multiple testing threshold for the pQTL and protein level validation was determined based on the number of available proteins, with an alpha value of 0.05.

### Gene-drug interaction effect of the targeting compounds on WMH and dementia-Pharmacogenomics:

Genetic heterogeneity contributes significantly to inter-individual variability in drug response^90–93^. To assess whether genetically predicted gene expression modifies the effect of drug classes on phenotypes of interest, we applied the TxEWAS framework^92^. This approach identifies gene-by-drug interactions using imputed gene expression and medication exposure data. To maximize sample size with available medication records, MRI data, and dementia status, we included individuals with documented use of medications grouped under pharmacological subgroups (ATC 4th level) of the substance (5th level under ATC classification) showing interaction with prioritized PCTs in the DGIdb analysis. Only unrelated individuals of European ancestry were included. Dementia status (ACD) was determined using ICD codes (see Supplementary Methods), excluding individuals diagnosed prior to the baseline medication assessment. WMH volumes were derived from UK Biobank Field ID 25781. Supplementary Table 10 provides an overview of drug classes analyzed, the number of individuals with phenotype data, and an indicator of whether the analysis of a given drug class is sufficiently powered for TxEWAS. Briefly, the TxEWAS method involves two steps: first, genetically predicted gene expression is imputed using GTEx v7 reference data; second, gene-by-drug interaction effects are tested using linear or logistic regression models, depending on the phenotype. Covariates included age, sex, birth year, Townsend deprivation index, and the first 16 genetic principal components. The variance of the effect size estimates is estimated with the robust sandwich variance estimator (SVE), to control for both heteroscedasticity^94^ and misspecification of the interaction variable, such as unmodeled dose-dependent effects^95,96^. Additional sensitivity analysis were conducted to assess potential endogeneity bias, as detailed elsewhere^97^.

#### Spatial colocalization of the PCTs in post-mortem brain tissues

*Bulk tissue analysis* - Leveraging bulk-tissue gene expression profiles from postmortem brain tissues of clinically and neuropathologically ascertained dementia cases with co-occurring vascular lesions (N = 22) and matched controls (N = 148) from the GTEx consortium (EUR) (see Supplementary Methods), we studied tissue-specific differential gene expression (DGE) of the prioritized drug-targets. A paired study design was implemented using DESeq2, estimating the main effects of brain region (DLPFC vs. cerebellum) and disease state (cases vs. controls), as well as their interaction effects. The model accounted for individual-level pairing by including subject IDs as a blocking factor. The resulting log2 fold change (Log2FC) estimated reflect whether gene expression levels differ due to the combined effect of brain region and disease state, relative to the baseline (cerebellum in control subjects). False discovery rate was controlled by implementing the Benjamini-Hochberg procedure. *Cell type specific analysis* - Furthermore, using spatial gene expression profiling (NanoString GeoMx^™^) from formalin-fixed paraffin-embedded (FFPE) tissue sections of the DLPFC (Brodmann area 9) from the NanoString Supplemental Cases obtained from the SuperAging Study, the 90 + Study (EUR) (see Supplementary Methods), we compared DGE of the prioritized drug-targets across cognitively normal individuals and dementia cases with increasing levels of cognitive impairment and co-existing chronic microinfarcts (mild cognitive impairment [MCI] with two microinfarcts, and dementia with three microinfarcts). DGE analyses were performed within fluorescently labeled cell types - astrocytes (GFAP), endothelial cells (CD31), and microglia (IBA1). We applied pairwise Wilcoxon rank-sum tests to compare Q3-normalized expression values between cognitively normal controls and the MCI and dementia groups, for each cell type. Benjamini-Hochberg correction was applied to control for multiple testing, with a significance threshold of *P* < 0.05.

### Targetable pathways enriched for WMH and its association with dementia outcomes:

*Pathway identification* - We first used the pharmagenic enrichment score (PES) pipeline^98^, to identify WMH-associated pathways that are potentially modifiable by pharmacological agents. Briefly, the PES pipeline implements the Multi-marker Analysis of GenoMic Annotation (MAGMA) framework to generate gene-level association statistics by aggregating SNP-level associations from WMH GWAS summary statistics. SNPs are mapped to protein-coding genes using a window of 5 Kb upstream and 1.5 Kb downstream, accounting for LD. To better capture the polygenic architecture of WMH, gene-level associations are calculated across multiple GWAS p-value thresholds (*P*_T_ < 0.5, *P*_T_ < 0.05, and *P*_T_ < 0.005). Next, using predefined druggable gene sets from the Molecular Signatures Database (MSigDB) - each including at least one gene targeted by an approved drug - a competitive gene-set enrichment test was performed at each *P*_T_ bin. This test evaluates whether genes within each pathway are more strongly associated with WMH than genes outside the set. Following previous recommendation^98^, a significance threshold of *P* < 0.001 was applied to identify enriched pathways. *PES score association with dementia outcomes* - We then assessed whether WMH-enriched, druggable pathways were also associated with dementia outcomes. For each significant pathway, we extracted SNPs within the genes comprising that pathway (from MSigDB) and computed individual-level PES (WMH-PES) scores in European participants from the UK Biobank diagnosed with AD, VaD, or ACD, along with cognitively health controls (see Supplemental Methods). Pathway-specific WMH-PES were calculated using PRSice^99^, as the weighted sum of LD-independent SNPs (r^2^ < 0.1), with weights derived from WMH GWAS effect sizes. Scores were standardized to reflect one SD changes. Associations between WMH-PES and dementia outcomes were tested at multiple *P*_T_ bins using binomial logistic regression, adjusting for age, sex, and the first 10 genetic principal components. To estimate risk stratification, individuals were grouped into quintiles based on their WMH-PES from the best-performing *P*_T_ model, and relative odds were calculated using the lowest quintile as the reference. Finally, pathway associations that were significant in the UK Biobank were replicated in two independent cohorts: the European-only HUNT study and a non-European Hispanic cohort (SALSA).

## Supplementary Files

This is a list of supplementary files associated with this preprint. Click to download.

- DrugrepurposecSVDAD070425SupplMethodsvF.docx
- DrugrepurposecSVDAD070425MAINCLEANFigTablesResSquarV.xlsx
- SupplementaryFigure1.docx

## Figures and Tables

**Table 1:** Targetable pathways enriched for WMH.

Pathway	No of Genes	Effect	StdErr	*P*value	*P* Threshold (*P*_T_)
Hypoxia pathway	26	0.250	0.068	1.30E-04	0.005
PAR4-mediated thrombin events	15	0.509	0.148	2.94E-04	0.5
P53 downstream effectors	72	0.144	0.042	3.04E-04	0.05
Coagulation pathway	63	0.145	0.044	4.80E-04	0.05
RhoA signaling pathway	44	0.410	0.128	6.76E-04	1
Matrisome pathway	946	0.064	0.020	8.90E-04	0.5

P threshold (*P*_T_) indicates SNPs below the association threshold in the WMH GWAS, contributing to the pathway enrichment analysis.

## Data Availability

The gene expression and eQTL datasets were collected from FHS (https://ftp.ncbi.nlm.nih.gov/eqtl/original_submissions/FHS_eQTL/ (complete unfiltered eQTL and annotation datasets) and publicly available GTEx project (https://www.gtexportal.org/home/). The GWAS of WMH trait is available at dbGAP (https://ftp.ncbi.nlm.nih.gov/dbgap/studies/phs002227/analyses/). The individual level phenotype data is available to authorized investigators through the UK Biobank portal (http://www.ukbiobank.ac.uk). Other datasets were previously published and available as mentioned in Methods.

## Code availability

Publicly available software and packages were utilized for this study: PLINK v.1.9 (https://www.cog-genomics.org/plink/1.9/), PLINK v.2.0 (https://www.cog-genomics.org/plink/2.0/), TxEWAS (https://github.com/michalsad/txewas_scripts), PRSice2 (https://choishingwan.github.io/PRSice/) and MAGMA (https://cncr.nl/research/magma/).
